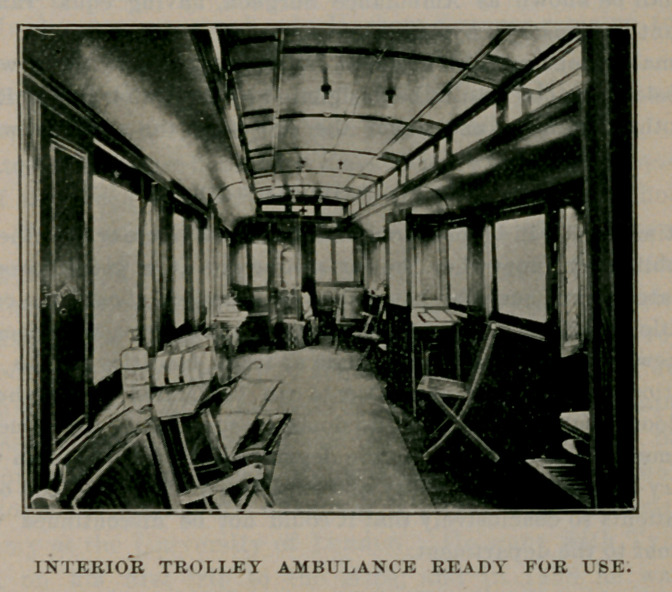# An Electric Railway Ambulance

**Published:** 1895-08

**Authors:** 


A Monthly Review of Medicine and Surgery.

*EDITORS:*

THOMAS LOTHROP, M. D. - - WM. WARREN POTTER, M. D.

All communications, whether of a literary or business nature, should be addressed to the managing editor: 284 FRANKLIN STREET, BUFFALO, N. Y.

VOL. XXXV. AUGUST, 1895. No. 1.

### AN ELECTRIC RAILWAY AMBULANCE.

WE HAVE heretofore, on several occasions, in these columns advocated the establishment in Buffalo of a trolley ambulance service. There are many reasons why this kind of an ambulance should be preferred to the present fashion of horse ambulances. In the first place it is a safer and more comfortable kind of conveyance for the sick and injured ; and, secondly, it is far safer for the citizens who, in a populous city like this, are in the greatest possible danger of being run over by the horse ambulances. Indeed, not long since one of our citizens was killed in this way. Moreover, horses attached to carriages are likely to be frightened by the noise and confusion incident to their rapid transit, and so, secondarily, they may do great damage to say nothing of the temporary embarrassment to traffic in our crowded thoroughfares. But there is still another cogent reason for the employment of the trolley ambulance. The first care of the wounded is an important office. If dressings be not applied then with aseptic skill, the surgeon who finally conducts the case may be greatly embarrassed in his combat with septic conditions, that might have been prevented by a carefully applied aseptic first dressing. It is not expected, of course, that these precautions can be obtained in the horse ambulance, but it is precisely what can be accomplished in the trolley ambulance. This latter is a hospital on wheels, with all the appointments of a modern surgical operating room. It is supplied with surgeons, nurses, dressings, sterilised water and everything that goes to make up a complete equipment for carrying out surgical attendance on the most modern basis of asepticism. There is no occasion for haste in transit, as every care can be given the wounded en route, even to feeding them.

*Reproduced from Harpers' Weekly.* *Copyright 1895 by Harper Brothers.*

INTERIOR VIEW OF TROLLEY AMBULANCE—SURGEON AND NURSES DRESSING THE WOUNDED.

In the right upper corner is an exterior view of the ambulance.

We need not carry the argument further at this time, for every point is more clearly presented by the engravings that are herewith published of the St. Louis ambulance car, that is in successful operation in that city. The small interior view was kindly loaned us by the *Medical Mirror*, Dr. I. N. Love, editor and owner. The large engraving was reduced from an illustration that appeared in *Harpers' Weekly* March 23, 1895, made by the kind permission of Messrs. Harper and Brothers, publishers, Franklin Square, New York.

We subjoin a description of the car taken from the *Medical Mirror*, March, 1895 :

DESCRIPTION OF CAR.

The total length of the car is 33 feet, including the platforms, the inside measurements being 24 feet long by 7 feet 2 inches wide. The platforms are about one-half the width of the car, and front the left half of the body ; the car being double-ended, the steps leading to the entrance, and not to the platform. The doors are located at the right hand side of the ends of the car, the steps being four in number, placed obliquely and affording access from either front or side with equal ease, this arrangement having been adopted to facilitate the exit or entrance of litter-bearers. A telescoping brass hand-rail guards the outer side of the steps, and these are so arranged that they can be lifted when not in use, thus preventing access to the car. The entrances are 5 feet, 2 inches in width, the doors moving on rollers placed at the top, and grooved below over a metal edge, secured to the sill, excluding cold and dust.

The car is lighted by seven windows on each side, 28x28 inches in size, and stationary, the lower half frosted. There are two drop sashes in each door, and a number of ventilators or adjustable deck lights above on the sides and ends.

The interior of the car is divided in the middle by a wooden partition 5 feet, 6 inches high, placed four inches from the floor, with two doors swinging either way in the center, one compartment being for males and the other for females. The partition is so constructed that both parts may be swung back if desired, and rest closely against the sides of the car. A drop desk is hinged to one side of the partition for the surgeon's use. Two hinged shelves are provided, attached to the sides of the car, on which to place dressings, instruments, etc.

To the right of each door on entering the car is placed a large locker for litters, blankets, towels and the like, while between the windows are eight small ones for papers, medicines, bandages, instruments, etc. Immediately back of the large lockers are two earth closet conveniences, (Heap's patent,) for both sexes.

Electric bells communicating with both platforms are placed near the middle of the car for the use of the surgeon. A lavatory is also located there, supplied with water by a pump from a tank under the floor, the metal work being silver plated, as are the water coolers at each end of the car.

The interior is finely finished in cherry, there being no spaces for dirt to accumulate. The floor is of quartered oak, double, with asbestos filling to deaden the sound. The ceiling is of bird's-eye maple in panels. All the trimmings are solid bronze, polished.

Lighting is secured by ten incandescent burners, sixteen candle power, four being placed in each compartment and one outside above

INTERIOR TROLLEY AMBULANCE READY FOR USE.

each platform. The car is warmed by six electric heaters placed along the sides just above the base board and occupying but little space, being 30x7½x3 inches in size.

The body of the car is painted white, trimmed with gold and blue. The lettering on the sides reads: "St. Louis Health Department Ambulance Car," and the last two words are repeated on each end. The red Geneva cross is displayed on the sides and the ends to emphasise the humane purposes to which the vehicle is devoted.

The car is mounted on two pivotal trucks of special construction, having two sets of equalising springs over the oil-box in addition to the springs that carry the car body. This truck will greatly reduce the noise while crossing other tracks and make it an easy-riding car.

Under the center of the body of the car a space is enclosed in which to carry stretchers, splints and the like; and a water tank and ice box are also provided under the car. The distance from the body to the rails is 3 feet, 3 inches. Draw bars are provided in case it should become necessary to tow the ambulance by means of another car. The car is equipped with an electric brake besides the usual hand brake, and arrangement is made for heating by electricity the water used in the lavatory.

The furnishings and equipment, supplied by the Health Department, consist of eighteen plain folding chairs, twelve having arms and six without arms, all provided with rubber fenders to prevent slipping and defacement of woodwork.

When in service it is proposed to man the car with a physician, who shall be known as Ambulance Surgeon, having equal rank with Assistant Dispensary Physician.

A male attendant with grade of day nurse is also to serve with the car, besides a motorman to be designated by the Union Depot Railroad Co., both in uniform, and under the orders of the surgeon while on duty.

RUN ON SCHEDULE TIME.

A time card will be prepared and published in order that the police and public may know when the car will be due at a given street or a designated point, enabling patients to be delivered aboard the car with but little handling or delay. This service, however, will necessarily be somewhat crippled until such time as track facilities to the several institutions are provided and patients can be landed under shelter at their doors ; but this want will no doubt be supplied in due time. The experimental service begun by the department early in autumn with an ordinary car, proved the utility and superiority of this method of handling patients so conclusively that it could not be discontinued without detriment to the department.

This car could be readily adapted to the necessities of Buffalo, where trolley lines run in all directions, by laying short pieces of track to the entrance of such hospitals as need them and by placing switches near railway stations and wharves.

This service, once established, would do away with all competition among the hospitals. The patient would be taken to the hospital selected by himself or his friends or by the proper public officer as the case might be ; or, perchance, to a private residence.

To establish this service into successful use it is requisite that there shall be coöperation between the mayor, department of health, the common council and the street railway managers. It is hoped that this will not be difficult to accomplish.

## Figures and Tables

**Figure f1:**
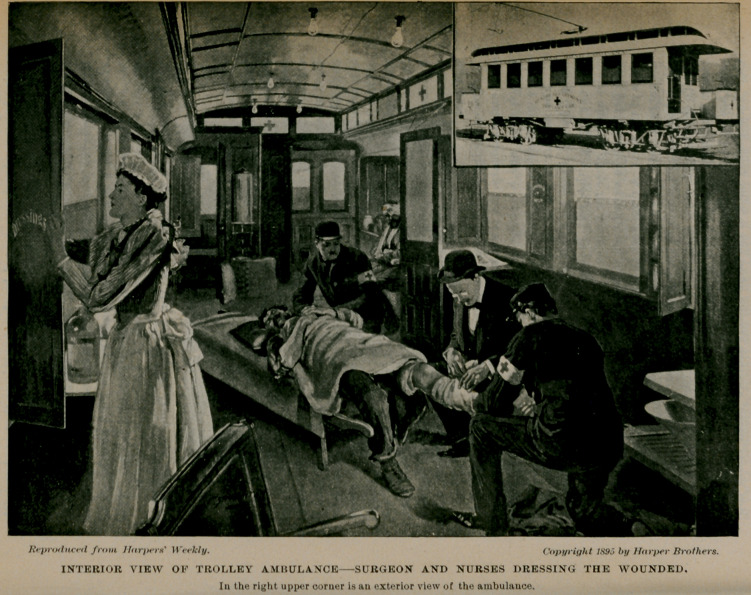


**Figure f2:**